# Structural correlates of impaired working memory in hippocampal sclerosis

**DOI:** 10.1111/epi.12193

**Published:** 2013-04-24

**Authors:** Gavin P Winston, Jason Stretton, Meneka K Sidhu, Mark R Symms, Pamela J Thompson, John S Duncan

**Affiliations:** * Epilepsy Society MRI Unit, Chesham LaneChalfont St Peter, United Kingdom; † Department of Clinical and Experimental Epilepsy, UCL Institute of NeurologyLondon, United Kingdom

**Keywords:** Temporal lobe epilepsy, Hippocampal sclerosis, Working memory, Voxel-based morphometry, Diffusion tensor imaging

## Abstract

**Purpose:**

Temporal lobe epilepsy (TLE) has been considered to impair long-term memory, whilst not affecting working memory, but recent evidence suggests that working memory is compromised. Functional MRI (fMRI) studies demonstrate that working memory involves a bilateral frontoparietal network the activation of which is disrupted in hippocampal sclerosis (HS). A specific role of the hippocampus to deactivate during working memory has been proposed with this mechanism faulty in patients with HS. Structural correlates of disrupted working memory in HS have not been explored.

**Methods:**

We studied 54 individuals with medically refractory TLE and unilateral HS (29 left) and 28 healthy controls. Subjects underwent 3T structural MRI, a visuospatial n-back fMRI paradigm and diffusion tensor imaging (DTI). Working memory capacity assessed by three span tasks (digit span backwards, gesture span, motor sequences) was combined with performance in the visuospatial paradigm to give a global working memory measure. Gray and white matter changes were investigated using voxel-based morphometry and voxel-based analysis of DTI, respectively.

**Key Findings:**

Individuals with left or right HS performed less well than healthy controls on all measures of working memory. fMRI demonstrated a bilateral frontoparietal network during the working memory task with reduced activation of the right parietal lobe in both patient groups. In left HS, gray matter loss was seen in the ipsilateral hippocampus and parietal lobe, with maintenance of the gray matter volume of the contralateral parietal lobe associated with better performance. White matter integrity within the frontoparietal network, in particular the superior longitudinal fasciculus and cingulum, and the contralateral temporal lobe, was associated with working memory performance. In right HS, gray matter loss was also seen in the ipsilateral hippocampus and parietal lobe. Working memory performance correlated with the gray matter volume of both frontal lobes and white matter integrity within the frontoparietal network and contralateral temporal lobe.

**Significance:**

Our data provide further evidence that working memory is disrupted in HS and impaired integrity of both gray and white matter is seen in functionally relevant areas. We suggest this forms the structural basis of the impairment of working memory, indicating widespread and functionally significant structural changes in patients with apparently isolated HS.

Working memory requires the maintenance and manipulation of information over short periods of time. Temporal lobe epilepsy (TLE) has been considered to impair formation and storage of long-term memories (Squire, 1992), whilst not affecting working memory (Cave & Squire, 1992). However, recent evidence suggests that working memory is compromised (reviewed in Stretton & Thompson, 2012).

Working memory can be explored by “n-back” paradigms employing progressively increasing working memory load (Braver et al., 1997). Functional MRI (fMRI) studies demonstrate activation in the frontal and parietal regions bilaterally (reviewed in Owen et al., 2005) that is reduced in subjects with epilepsy (Vlooswijk et al., 2011; Stretton et al., 2012). Evidence for hippocampal involvement in working memory is mixed, as fMRI studies have shown both task-dependent hippocampal activation (Axmacher et al., 2009) and deactivation (Cousijn et al., 2012). We have recently found in patients with hippocampal sclerosis (HS) that deactivation of the ipsilateral hippocampus with increasing working memory load is disrupted and associated with impaired working memory performance (Stretton et al., 2012).

Manipulation of information in working memory is an active process requiring executive regulation and controlled attention. It involves a complex neural network in which interconnecting white matter is critical. Aging and disease processes are hypothesized to disrupt working memory through cortico-cortical and cortico-subcortical disconnection. Structural connectivity can be explored using diffusion tensor imaging (DTI), which noninvasively assesses the integrity of white matter. Fractional anisotropy (FA) quantifies the degree of directionality of water diffusion and typically falls in disease processes (Beaulieu, 2002), whereas mean diffusivity (MD), which quantifies the overall degree of diffusion of water molecules, increases.

Maturation of the gray and white matter of the frontoparietal network is seen in healthy children (Olesen et al., 2003) and is related to working memory performance (Nagy et al., 2004; Ostby et al., 2011). Changes within white matter connections between frontal and parietal regions, including the superior longitudinal fasciculus (SLF) and cingulum, correlate with working memory decline in normal aging (Kennedy & Raz, 2009; Charlton et al., 2010), multiple sclerosis (Dineen et al., 2009), schizophrenia (Karlsgodt et al., 2008), and traumatic brain injury (Palacios et al., 2011). White matter changes in the parietal lobe consistent with plasticity have been observed following working memory training (Takeuchi et al., 2010).

We previously identified reduced activation of the right superior parietal lobe and impaired deactivation of the ipsilateral hippocampus in a cohort of patient with HS undergoing fMRI studies (Stretton et al., 2012), but the structural correlates remain unexplored in this population. A reasonable hypothesis is that the frontoparietal pathways affected in other conditions are involved in the working memory impairment observed in HS. A meta-analysis of DTI studies identified widespread bilateral changes within white matter pathways in TLE including the cingulum (Otte et al., 2012), although changes in the SLF were less consistent. The structural integrity of the temporal lobe itself in working memory has not been considered.

The present study investigates structural changes within the working memory network in a large cohort of patients with HS (including the previous cohort) to address the following hypotheses:

1. Structural changes exist in the gray matter of the bilateral frontoparietal working memory network identified by fMRI and in white matter tracts connecting these regions.
2. Working memory performance correlates with the structural changes in the frontoparietal network.
3. Working memory involves and is affected by the structural integrity of the temporal lobes.

We report fMRI findings in this larger cohort to enable comparison between the regions identified with functional and structural changes.

## Materials and Methods

### Subjects

We studied 54 individuals with medically refractory TLE and unilateral HS (29 left; age range 18–56 years, median 42 years, 21 male) and 28 healthy age-matched controls (age range 19–64 years, median 37 years, 11 male) without any history of neurologic or psychiatric disease (Table 1). Individuals with TLE were undergoing presurgical evaluation at the National Hospital for Neurology and Neurosurgery, London including structural MRI scans at 3 T, video–electroencephalography (EEG) and neuropsychology. Investigations confirmed seizure onset within the ipsilateral medial temporal lobe and excluded contralateral hippocampal pathology on qualitative and quantitative MRI criteria. The study was approved by the National Hospital for Neurology and Neurosurgery and the Institute of Neurology Joint Research Ethics Committee, and written informed consent was obtained from all subjects.

**Table:** Clinical and demographic characteristics

	Left HS (n = 29)	Right HS (n = 25)	Healthy controls (n = 28)
Gender (male/female)	15/14	6/19	11/17
Handedness (left/right)	5/24	4/21	3/25
	Median (IQR)	Median (IQR)	Median (IQR)
Age (years)	40 (16)	42 (14)	37 (22)
Age at seizure onset (years)	13 (14)	9 (15)	n/a
Duration of epilepsy (years)	26 (22)	29 (23)	n/a
Seizures frequency (per month)	6 (12)	5 (10)	n/a

### Magnetic resonance data

MRI studies were performed on a 3 T GE Signa HDx scanner (General Electric, Milwaukee, Wisconsin, U.S.A.) using a body coil for transmission, and eight channel phased array coil for reception. Standard imaging gradients with a maximum strength of 40 mT/m and slew rate 150 T/m/s were used. Clinical sequences performed included a coronal T1-weighted volumetric acquisition with 170 contiguous 1.1 mm thick slices (matrix 256 × 256, in-plane resolution 0.9375 × 0.9375 mm) used for the voxel-based morphometry (VBM) analysis.

For the working memory fMRI task, gradient-echo planar T2*-weighted images were obtained covering the whole brain with 50 oblique axial 2.4 mm slices (0.1 mm gap), SENSE factor 2, field of view 24 cm, 64 × 64 matrix giving in-plane resolution of 3.75 × 3.75 mm. Echo time (TE) was 25 msec and repetition time (TR) was 2.5 s.

DTI data were acquired using a cardiac-triggered single-shot spin-echo planar imaging (EPI) sequence with TE 73 msec. Sets of 60 contiguous 2.4-mm thick axial slices were obtained covering the whole brain with diffusion sensitizing gradients applied in 52 noncollinear directions (*b*-value 1,200 s/mm^2^ [δ = 21 msec, Δ = 29 msec, using full gradient strength of 40 mT/m]) along with six nondiffusion weighted scans. Gradient directions were calculated and ordered as described elsewhere (Cook et al., 2007). Field of view was 24 cm, acquisition matrix size was 96 × 96 zero filled to 128 × 128 to give a reconstructed voxel size of 1.875 × 1.875 × 2.4 mm.

### Working memory fMRI paradigm

The working memory network was identified using a modified version of the “*n*-back” task (Callicott et al., 1999; Kumari et al., 2003) in which subjects were required to monitor the location of dots within a diamond shaped box on the screen at a given delay with the original occurrence (0-, 1- or 2-back). The presentation paradigm and analysis using Statistical Parametric Mapping (SPM8) software (http://www.fil.ion.ucl.ac.uk/spm/) has been previously described (Stretton et al., 2012). Individual subject images for the “2-back” minus “0-back” contrast were compared in an analysis of variance (ANOVA) with group (control, left HS, right HS) as a factor to examine the main effects and to highlight regions demonstrating more or less activation in one group compared to another.

### Voxel-based morphometry

T1-weighted images were segmented into gray matter, white matter, and cerebrospinal fluid using New Segment in SPM8. The Diffeomorphic Anatomical Registration using Exponentiated Lie algebra (DARTEL) framework was used to produce a group template to which all subjects were registered (Ashburner, 2007). Gray matter segmentations were normalized to Montreal Neurological Institute (MNI) space applying modulation and smoothing with a Gaussian kernel of 8 mm full-width at half-maximum to produce maps of gray matter volume (GMV). Total intracranial volumes were determined using a locally written script and included in all analyses to correct for the effects of brain size. A mask produced by thresholding the group gray matter template at 0.2 was used to confine analyses to gray matter.

ANOVA was performed to determine group differences in GMV. Further analysis was performed in the LHS and RHS patient subgroups to determine the relationship of GMV to a global measure of working memory (see below, threshold of p = 0.001 uncorrected, minimum cluster size of 10).

### DTI processing and analysis

Eddy current correction of the data was performed using eddy_correct in FSL (http://www.fmrib.ox.ac.uk/fsl/), and maps of FA and MD were generated for each patient using the FMRIB Diffusion Toolbox. The FA maps were aligned to the FMRIB58 template using nonlinear registration within the TBSS framework and the same transformation was applied to the MD maps.

The resulting images were smoothed using an 8 mm full width half maximum (FWHM) Gaussian kernel. A mask produced by thresholding the FMRIB58 template with an FA value of 0.2 was used to confine analyses to white matter. Group comparisons of LHS or RHS groups versus controls were performed using SPM8 (threshold of FWE p < 0.05), and regression analyses were performed in each subgroup to determine where diffusion measures correlated with a global measure of working memory (below, threshold of 0.001 uncorrected, minimum cluster size of 10). In view of the previously strong correlations observed between diffusion measures and age (Charlton et al., 2010), age was included as a covariate in all analyses.

Finally, the mean FA of the entire white matter skeleton produced by the TBSS framework was determined for each subject. The same measures were derived for the two key white matter tracts connecting the frontal and parietal lobes previously implicated in working memory (superior longitudinal fasciculus, SLF; cingulum) and one control tract (corticospinal tract, CST) using a mask derived from the Johns Hopkins University white matter tractography atlas. The correlation between these diffusion measures and working memory performance was determined using a one-tailed *t*-test with the hypothesis that reduced FA would correlate with impaired performance.

### Neuropsychological measures of working memory

Three working memory span tasks were administered to assess working memory capacity (see Stretton et al., 2012). The digits backwards condition from the WAIS-III Digit Span subtest was used to measure verbal working memory, whereas the Gesture Span task and the Motor Sequences task were used as measures of spatial span.

To explore the relationship between GMV, white matter integrity, and working memory performance, and to avoid multiple comparisons, a single measure of working memory was derived using a principal component analysis (PCA) in PASW v18 (SPSS, Chicago, IL, U.S.A.). The scores from the three out-of-scanner span tasks were combined with the in-scanner performance (percentage correct) on the most demanding task, the 2-back condition.

A single component with eigenvalue >1 was found, which explained 65% of the variance. Seven subjects (four left HS, three right HS) were excluded from the PCA and subsequent correlation analyses due to incomplete data through an inability to understand the instructions necessary to perform the fMRI paradigm. This component representing an overall measure of global working memory capacity was used for the correlation analyses.

## Results

### Working memory performance

Individuals with LHS or RHS both performed significantly less well than healthy controls on measures of working memory (Table 2, one-way ANOVA for each measure, p < 0.005). The working memory PCA score significantly differed between the patient groups and controls (F_2,72_ = 14.9, p < 0.001).

**Table:** Working memory scores, with means and standard deviations

Measure	Controls	L HS	R HS
Digit span backwards	4.61 (1.17)	3.41 (1.12)	3.21 (0.93)
Gesture span	3.14 (0.62)	2.42 (0.69)	2.71 (1.00)
Motor sequencing	6.07 (2.24)	4.12 (2.03)	3.87 (2.21)
2-back% correct	71.6% (20.5%)	48.4% (20.4%)	54.2% (23.4%)
Working memory PCA	0.70 (0.92)	−0.41 (0.72)	−0.42 (0.90)

### Working memory fMRI

The multiple-item working memory network comprising the bilateral middle frontal gyrus (MFG) and superior parietal lobe (SPL) was identified using the 2-back minus 0-back contrast (Fig. S1A). Both LHS and RHS groups showed significantly less activation in the right SPL compared to controls (Fig. S1B, conjunction analysis, z = 3.32, uncorrected p < 0.001) confirming our previous finding in a smaller group (Stretton et al., 2012). No significant differences were identified in the frontoparietal network activation between LHS and RHS.

### Voxel-based morphometry

**Figure 1:**
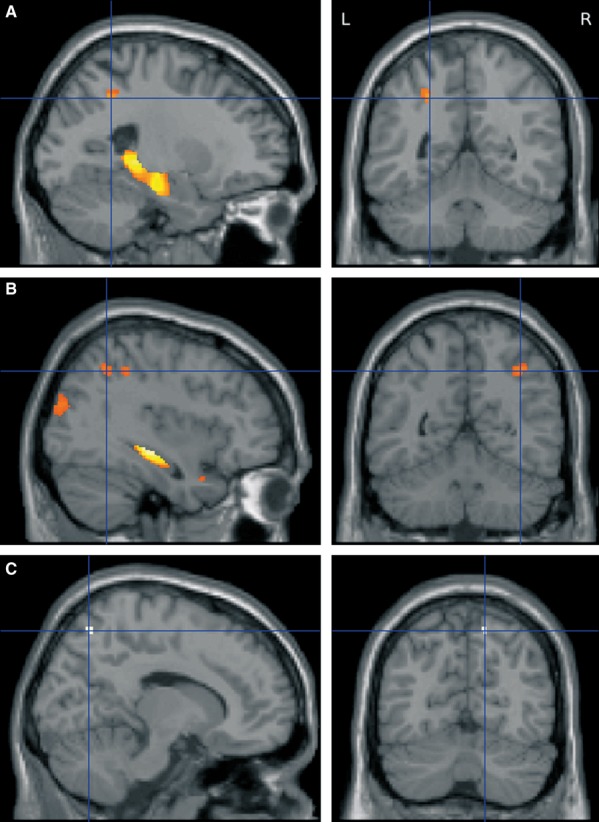
(A) In LHS, GMV loss was observed in the left hippocampus and left parietal lobe (uncorrected p = 0.001). (B) In RHS, GMV loss occurred in the right hippocampus, right parietal lobe, and other parts of the right hemisphere (uncorrected p = 0.001). (C) Working memory performance correlated with GMV of the right SPL in LHS (uncorrected p = 0.001).

Group comparisons between healthy controls and patients with LHS or RHS identified GMV loss confined to the left and right hippocampus, respectively (FWE p = 0.05). At a lower threshold (uncorrected p = 0.001, minimum cluster size 10), GMV loss was also seen in the ipsilateral parietal lobe in both patient groups (Fig. 1A,B). In the RHS subgroup, additional areas of GMV loss were observed throughout the right hemisphere (superior frontal, superior, and middle temporal gyri, and occipital region) and in the caudate nucleus bilaterally (Table S1). A comparison of the atrophy between LHS and RHS groups revealed the respective hippocampi, and additionally in RHS there were areas of atrophy within the right hemisphere, including the MFG and parietal lobe compared to LHS (uncorrected p = 0.001, minimum cluster size 10).

In LHS, working memory performance correlated with GMV in the right SPL (Fig. 1C, uncorrected p = 0.001, minimum cluster size 10). In RHS, working memory performance correlated with GMV in the left and right frontal lobes, left posterior temporal lobe, precuneus, and cerebellum (Table S2).

### Voxel-based analysis of DTI

The group comparison revealed reduced FA predominantly within the left temporal lobe and limbic system but also within the left inferior frontal, cuneus, and cerebellum in LHS compared to controls. FA was reduced in the right temporal/limbic region, right middle frontal, precuneus, and cerebellum in RHS compared to controls. These findings are not reported further as they are comparable to our previous report on this topic (Focke et al., 2008).

**Figure 2:**
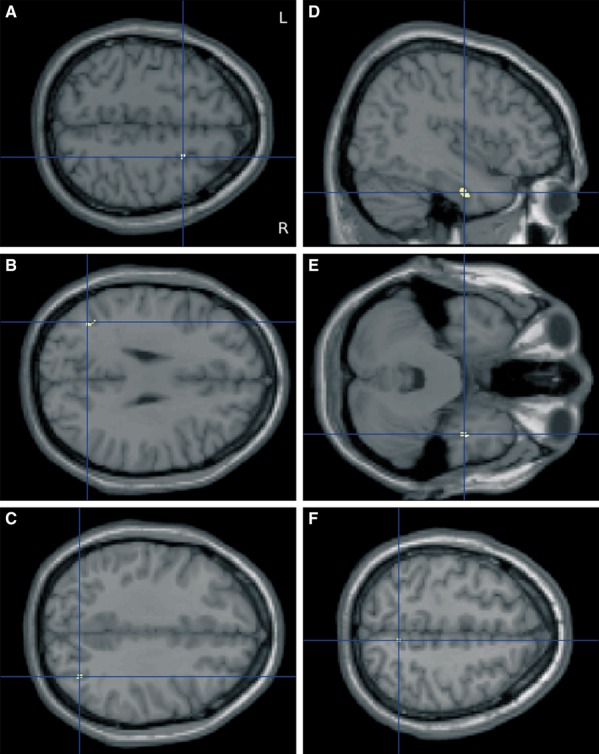
In LHS, better working memory performance correlated with higher FA in the frontoparietalworking memory network (right MFG, A; bilateral parietal lobe, B and C), the right inferior temporal lobe (D and E), and the right cingulum (F). Epilepsia © ILAE

**Figure 3:**
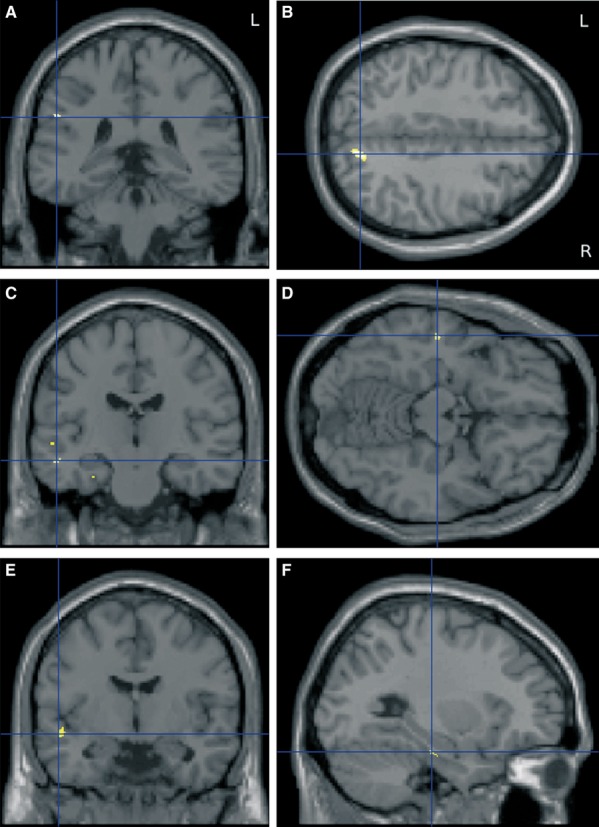
In RHS, better working memory performance correlated with higher FA in the left parietal lobe (A), right cingulum (B), several regions of the left temporal lobe (C–F), and the cerebellum (not shown).

In healthy controls, working memory correlated with higher FA in three small clusters within the left SFG and right inferior temporal gyrus (Table S3). In the LHS subgroup, correlations were observed in the frontoparietal working memory network—the right MFG and bilaterally in the parietal lobe at the extremities of the SLF—and also in the right cingulum and right inferior temporal lobe (Fig. 2). In RHS, working memory correlated with higher FA in the left parietal lobe, right cingulum, and several areas within the left temporal lobe—superior, middle, and parahippocampal gyri—and cerebellum (Fig. 3).

**Figure 4:**
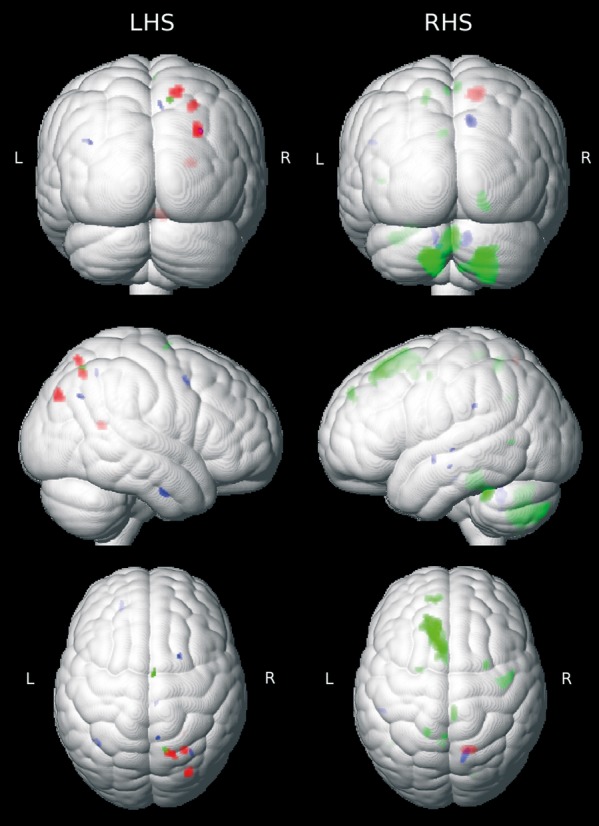
Summary of changes observed in the working memory network observed in LHS and RHS, indicating areas of reduced activation in the 2-back minus 0-back contrast for the working memory fMRI task (red); areas where the degree of gray matter atrophy correlated with working memory performance (green); and areas where working memory performance correlated with FA (blue). Epilepsia ILAE

In LHS, lower MD within the frontoparietal working memory network—left SFG and bilateral SPL at either end of the SLF—and the right cingulum and orbitofrontal cortex correlated with better working memory performance (Fig. S2 and Table S4). No significant negative correlations were observed in healthy controls or RHS.

### Tract-based analysis of DTI

Mean FA of the TBSS skeleton was significantly reduced in both LHS (0.421, p = 0.001) and RHS (0.421, p < 0.001) compared to healthy controls (0.441). In the LHS subgroup, a single outlier with mean FA three standard deviations below the group mean was excluded from subsequent analyses.

In LHS, working memory performance correlated with mean FA of the left SLF (one-tailed p = 0.013), right SLF (p = 0.021), left cingulum (p = 0.019), and right cingulum (p = 0.025). No correlations were observed with the mean FA of either corticospinal tract, or in the control or RHS subgroups. None of these correlations remained significant following a Bonferroni correction for multiple comparisons. Working memory performance correlated with the mean FA of the entire TBSS skeleton (p = 0.021) in LHS, but not in healthy controls or RHS.

## Discussion

### Summary of results

A bilateral frontoparietal working memory network was delineated in controls and patients with HS using fMRI with reduced activation of the right parietal lobe seen in LHS and RHS. Both VBM and DTI indicated the effect of TLE on this frontoparietal network, especially the parietal lobe, and suggested a role of the temporal lobe contralateral to the seizure focus in the maintenance of working memory performance.

In LHS, gray matter loss was seen within the ipsilateral hippocampus and parietal lobe, with the maintenance of the GMV of the contralateral parietal lobe associated with better performance. DTI demonstrated that white matter connections within the frontoparietal network—the extremities of the SLF bilaterally and the right cingulum—and the integrity of the contralateral temporal lobe were important for working memory performance.

In RHS, gray matter loss was also seen in the ipsilateral hippocampus, parietal lobe, and other parts of this hemisphere. The structural integrity of the frontoparietal network was related to performance, as working memory correlated with GMV in both frontal lobes and the precuneus and FA of the left parietal lobe and right cingulum. Both GMV and FA of the contralateral temporal lobe were related to performance.

### Impaired working memory in TLE

Working memory is crucial for daily life and is dependent on distributed neural networks including bilateral frontoparietal brain regions. Our data provide further evidence that working memory is disrupted in TLE (reviewed in Stretton & Thompson, 2012).

### Frontoparietal working memory network

The frontoparietal working memory network comprises strongly interconnected anterior (dorsolateral and ventrolateral prefrontal) and posterior (parietal cortex) regions (D’Esposito, 2008), which undergo combined functional and structural maturation (Klingberg et al., 2002). Areas adjoining the superior frontal sulcus are consistently activated during both visuospatial and nonspatial working memory tasks (reviewed in Klingberg, 2006) and are required for the voluntary control of attention, whereas the superior parietal lobe is involved in continuous updating of information (Owen et al., 2005) and manipulation of information within working memory (Koenigs et al., 2009).

Cortical thickness in these regions is related to verbal working memory performance following traumatic brain injury (Wilde et al., 2011), and frontal and parietal lesion burden correlates with attention and verbal working memory in multiple sclerosis (Sperling et al., 2001). Reduced functional (Liao et al., 2011) and structural (Riley et al., 2010) frontoparietal connectivity has been demonstrated in TLE. Our data confirm our previous finding of reduced fMRI activation of the parietal lobe in both LHS and RHS. This was in the right hemisphere for both patient groups, in keeping with the right parietal dominance of the spatial working memory task employed (Smith & Jonides, 1998; Owen et al., 2005).

### Changes in frontoparietal gray matter

VBM demonstrated GMV loss within the ipsilateral parietal lobe in both patient groups, and in LHS the integrity of gray matter in the contralateral parietal lobe was associated with better working memory, implying an important role for this part of the brain. The more widespread correlations observed in RHS may reflect the increased sensitivity of the working memory performance measure to seizures arising from the right hemisphere in view of the right dominance of the spatial working memory task.

Cortical thinning has been reported in patients with TLE, both HS and nonlesional, in areas that include the parietal lobe bilaterally (Labate et al., 2011). Furthermore, a meta-analysis of VBM studies in TLE showed that bilateral parietal atrophy was commonly observed (Keller & Roberts, 2008).

All the studies in this meta-analysis used SPM2 or earlier, a 1.5 T or 2 T scanner, and some involved heterogeneous populations, whereas the present study includes a homogeneous population of patients with HS, data acquired on a 3 T scanner, and employs the DARTEL registration framework (Ashburner, 2007) included within the latest version of SPM. This registration framework was developed to improve intersubject registration and iteratively generates a group-specific template from the subjects. The templates generated, however, do not include the thalamus. The present study detected fewer regions of altered GMV than the meta-analysis, particularly in LHS. This may reflect increased sensitivity from the meta-analysis pooling several studies, some of which include a greater number of participants, particularly controls, than the present study.

### Voxel-based changes in frontoparietal white matter

The frontal and parietal cortices are connected by two major white matter pathways. The SLF is the primary direct pathway connecting the frontal and parietal cortices and runs between the MFG/dorsolateral prefrontal cortex and the supramarginal gyrus in the inferior parietal lobe. The cingulum forms part of the limbic system, runs within the cingulate gyrus around the corpus callosum, and connects the prefrontal cortex and parietal lobes, thus playing a role in working memory (Kondo et al., 2004), and continues inferiorly to terminate in the anteromedial temporal lobe. The anterior cingulate is thought to be required for executive control of attention and performance monitoring (Barch et al., 1997; Carter et al., 1998), whereas the posterior cingulate mediates spatial attention and orientation (Hirono et al., 1998; Small et al., 2003).

In children, development of verbal working memory correlates with FA of the left SLF and cingulum (Ostby et al., 2011) and development of spatial working memory correlates with FA at the extreme of the SLF adjacent to the left superior frontal sulcus (Nagy et al., 2004). Diffusion characteristics of the SLF predict spatial working memory performance in children (Vestergaard et al., 2011) and verbal working memory in adults (Burzynska et al., 2011). Working memory training promotes plasticity with changes observed around the inferior parietal sulcus, body of the corpus callosum, and in the frontoparietal region (Takeuchi et al., 2010).

Frontoparietal white matter changes have also been observed in disease. Following traumatic brain injury, working memory performance correlates with FA in the left frontal region and cingulum (Wilde et al., 2011), and loss of integrity of the left SLF in recent onset schizophrenia is associated with impaired verbal working memory (Karlsgodt et al., 2008). In multiple sclerosis, working memory performance correlates with integrity of SLF, cingulum, forceps major, and body and splenium of the corpus callosum (Dineen et al., 2009).

In LHS, we found that working memory performance correlated with diffusion characteristics of the frontal and parietal white matter bilaterally. The regions identified lie adjacent to the gray matter of the working memory network at the extremes of the SLF bilaterally and also within the right cingulum. In RHS, fewer areas were observed including the left parietal lobe and right cingulum. This supports the involvement of these two tracts in patients with TLE and the interpretation that impaired frontoparietal connectivity impacts on working memory performance. The differences between the groups are compatible with previous data showing more extensive and bilateral white matter changes in LHS than RHS (Ahmadi et al., 2009) with the left hemisphere postulated to be more vulnerable to early insults as it matures more slowly than the right hemisphere (Kemmotsu et al., 2011).

### Tract-based changes in frontoparietal white matter

In view of the importance of the SLF and cingulum, we explored the correlation between their integrity and working memory performance. Although working memory performance correlated with the FA of the SLF and cingulum bilaterally in LHS, interpretation must be cautious, as working memory performance also correlated with the mean FA of the entire white matter skeleton and these correlations do not remain significant after correction for multiple comparisons. In normal aging, an age-related fall in whole brain FA and rise in MD correlated with declining working memory (Charlton et al., 2006). Similarly, impaired working memory following traumatic brain injury correlated with global mean FA making any tract-specific correlations less specific (Palacios et al., 2011).

Changes along white matter tracts do not occur uniformly. Maps of diffusion parameters along the length of the uncinate, arcuate, and inferior longitudinal fasciculi in patients with drug-resistant TLE demonstrate that these parameters may be changed in only part of the tracts with an emphasis on the extremities (Bernasconi et al., 2012). It is noteworthy that the changes observed, particularly within the LHS population, occur at either end of the SLF and so any whole tract–based measures may fail to observe the full extent of these more local effects.

### The role of the temporal lobe

Two main hypotheses have been postulated for the disruption of working memory in TLE. Firstly, propagation of epileptic activity from the epileptogenic zone to eloquent cortex responsible for working memory (Hermann et al., 1988; Vlooswijk et al., 2011). Secondly, involvement of temporal structures, particularly the hippocampus, in the working memory network (Corcoran & Upton, 1993).

The evidence for hippocampal involvement is conflicting. Initial reports suggest working memory is hippocampal-dependent with activation during different working memory functions, including encoding (Karlsgodt et al., 2005; Mainy et al., 2007), maintenance (Axmacher et al., 2007), and retrieval (Schon et al., 2009). Recently, however, there has been increasing recognition of the role of hippocampal deactivation. In healthy volunteers, bilateral hippocampal deactivation occurs (Astur & Constable, 2004; Astur et al., 2005) and progressive deactivation with increasing working memory load (Cousijn et al., 2012) is relevant to task performance (Hampson et al., 2006). In TLE, we found deactivation of the ipsilateral hippocampus is disrupted and related to working memory performance (Stretton et al., 2012).

In our subjects, GMV of the left posterior temporal region correlated with working memory performance in RHS. FA of the right inferior temporal lobe was related to performance in both healthy controls and LHS, whereas in RHS the correlations were observed in the left temporal lobe. Contralateral hippocampal deactivation was preserved in both LHS and RHS (Stretton et al., 2012), and the structural integrity of the contralateral temporal lobe appears important in working memory performance. Together, these suggest that the temporal lobe plays a key role in the working memory network.

### Strengths and limitations

This study included a large homogeneous population of patients with TLE and HS who had both functional and structural imaging. Three modalities were employed to provide complementary information on both the gray matter (fMRI, VBM) and white matter (DTI) changes underlying impaired working memory in TLE. A single measure of working memory was derived to avoid the problems of multiple comparisons inherent in many studies. It does, however, combine both verbal and nonverbal measures of working memory and there is some evidence for material-specific lateralization (Smith & Jonides, 1998).

For VBM, the latest and most accurate form of intersubject image coregistration, DARTEL, was used to maximize accuracy, which in combination with a smaller group size and 3 T acquisition may explain some of the differences observed in comparison to previous literature. For DTI, although the TBSS framework was used to coregister images, a voxel-based approach was subsequently employed to provide whole brain coverage. This technique was chosen to ensure the detection of changes not just confined to the white matter skeleton. This is important in light of recent data suggesting that tracts are not uniformly affected in TLE and many of the changes detected lie at the extremities of the white matter tracts in proximity to the gray matter regions subserving working memory functions. All voxel-based analyses are, however, subject to limitations of the accuracy of image coregistration, and results can vary with the choice of smoothing kernel size (Jones et al., 2005; Smith et al., 2006).

Changes in diffusion parameters are nonspecific and could reflect a variety of underlying neuropathologic changes, including the geometry and organization of axons, degree of myelination, and axonal diameter, density, and spacing (Basser & Pierpaoli, 1996; Beaulieu, 2002). Although DTI data have previously been combined with magnetic resonance spectroscopy to demonstrate axonal loss (Charlton et al., 2006), the spatial resolution is poor. Current developments in DTI could disentangle the contribution by different factors to alterations in FA.

## Conclusion

This study provides evidence that working memory is affected in TLE and that there is impaired integrity of both gray and white matter in functionally relevant areas. We suggest that this forms the structural basis of the impairment of working memory in TLE, indicating widespread and functionally significant structural changes in patients with apparently isolated HS.

A cross-sectional study cannot determine whether these changes arose at the time of or before the onset of epilepsy or are a result of ongoing seizure activity. We are conducting longitudinal studies of medically and surgically treated patients to address this crucial point. Further work combining functional and structural connectivity analyses of the temporal lobe will further explore the role of the temporal lobe in working memory.

## Supporting Information

**Table S1.** Regions of GMV loss in patients compared to healthy controls (uncorrected p = 0.001, 10 voxel clusters = 33.75 mm^3^). **Table S2.** Regions where GMV correlated with working memory performance (uncorrected p = 0.001, 10 voxel clusters = 33.75 mm^3^). **Table S3.** Regions where FA correlated with working memory performance (uncorrected p = 0.001, age as covariate, 10 voxel clusters = 10 mm^3^). **Table S4.** Regions where MD correlated with working memory performance (uncorrected p = 0.001, age as covariate, 10 voxel clusters = 10 mm^3^).

Figure S1 (**A**) A bilateral frontoparietal working memory network was identified using the 2-back minus 0-back contrast in all subjects. (**B**) In both LHS and RHS groups there was significantly less activation in the right SPL compared to controls (uncorrected p = 0.001, conjunction).

Figure S2 In LHS, better working memory performance correlated with lower MD in the frontoparietal working memory network (left SFG, A and B; bilateral SPL, A and C), the right cingulum (D), and the right orbitofrontal cortex (not shown).
