# Supplementary material for: Structural correlates of impaired working memory in hippocampal sclerosis
Source: Epilepsia. 2013 Apr 24;54(7):1143–53. doi: 10.1111/epi.12193 (PMC3806272; doi:10.1111/epi.12193)
Supplement: Supplementary file 1 — Table S1. Regions of GMV loss in patients compared to healthy controls (uncorrected p = 0.001, 10 voxel clusters = 33.75 mm3). Table S2. Regions where GMV correlated with working memory performance (uncorrected p = 0.001, 10 voxel clusters = 33.75 mm3). Table S3. Regions where FA correlated with working memory performance (uncorrected p = 0.001, age as covariate, 10 voxel clusters = 10 mm3). Table S4. Regions where MD correlated with working memory performance (uncorrected p = 0.001, age as covariate, 10 voxel clusters = 10 mm3). [file epi0054-1143-sd1.docx]

**Supplementary Table 1**. Regions of GMV loss in patients compared to healthy controls (uncorrected p=0.001, 10 voxel clusters = 33.75mm^3^)

*GMV: Left HS < Controls*

| **Region** | **Cluster size (mm^3^)** | **Z score** | **MNI coordinates** |
| --- | --- | --- | --- |
| Left hippocampus | 6770 | 6.98 | -30,-30,-5 |
| Left inferior parietal lobe | 496 | 3.97 | -24,-51,42 |
| Left occipital lobe (calcarine) | 199 | 3.48 | -18,-103,-6 |

*GMV: Left HS > Controls*

| **Region** | **Cluster size (mm^3^)** | **Z score** | **MNI coordinates** |
| --- | --- | --- | --- |
| Right anterior cingulated | 64 | 3.32 | 10,20,27 |
| Right parietal lobe (postcentral) | 44 | 3.25 | 12,-37,78 |

*GMV: Right HS < Controls*

| **Region** | **Cluster size (mm^3^)** | **Z score** | **MNI coordinates** |
| --- | --- | --- | --- |
| Right hippocampus | 6318 | 7.21 | 33,-25,-6 |
| Right occipital lobe (calcarine) | 1823 | 4.49 | 15,-97,3 |
| Right middle temporal gyrus (posterior) | 2157 | 4.44 | 52,-64,7 |
| Right middle frontal gyrus | 1286 | 4.09 | 24,-12,52 |
| Right superior temporal gyrus (anterior) | 577 | 4.09 | 33,9,-30 |
| Right caudate | 520 | 3.75 | 15,6,21 |
| Right occipital lobe (superior) | 1944 | 3.73 | 24,-93,27 |
| Right superior temporal gyrus (posterior) | 263 | 3.72 | 56,-34,19 |
| Right inferior parietal lobe | 1073 | 3.67 | 42,-46,49 |
| Right middle temporal gyrus | 830 | 3.59 | 60,-37,1 |
| Right occipital lobe (inferior occipital gyrus) | 543 | 3.56 | 33,-82,-11 |
| Right occipital lobe (calcarine) | 250 | 3.52 | -9,-73,10 |
| Right middle temporal gyrus (posterior) | 429 | 3.38 | 52,-72,25 |
| Right posterior cerebellum | 290 | 3.38 | 8,-67,-35 |
| Left caudate | 203 | 3.37 | -15,6,19 |
| Left occipital lobe (BA18) | 57 | 3.21 | -20,-103,-5 |
| Right precentral gyrus | 41 | 3.19 | 51,-12,45 |
| Left occipital lobe (calcarine) | 34 | 3.18 | -6,-96,4 |

*GMV: Right HS > Controls*

| **Region** | **Cluster size (mm^3^)** | **Z score** | **MNI coordinates** |
| --- | --- | --- | --- |
| None |  |  |  |

**Supplementary Table 2**. Regions where GMV correlated with working memory performance (uncorrected p=0.001, 10 voxel clusters = 33.75 mm^3^)

*Left HS*

| **Region** | **Cluster size (mm^3^)** | **Z score** | **MNI coordinates** |
| --- | --- | --- | --- |
| Right SFG (SMA) | 49 | 3.33 | 3,-6,72 |
| Right superior parietal lobe | 51 | 3.25 | 14,-66,57 |
| Left SFG (inferior) | 47 | 3.24 | -24,51,-20 |

*Right HS*

| **Region** | **Cluster size (mm^3^)** | **Z score** | **MNI coordinates** |
| --- | --- | --- | --- |
| Left frontal (medial SFG) | 4384 | 4.05 | -9,30,52 |
| Posterior cerebellum | 6649 | 3.88 | 0,-75,-32 |
| Left cerebellum (fusiform) | 2376 | 3.74 | -22,-34,-23 |
| Right frontal (MFG) | 98 | 3.61 | 30,2,66 |
| Right frontal (precentral) | 1121 | 3.60 | 47,-9,42 |
| Right cerebellum (inferior) | 4590 | 3.59 | 24,-73,-51 |
| Right frontal (paracentral lobule) | 257 | 3.55 | 4,-36,66 |
| Left precuneus | 64 | 3.48 | -2,-55,63 |
| Left precuneus | 182 | 3.48 | -9,-60,34 |
| Left precuneus | 263 | 3.46 | -18,-51,55 |
| Left frontal (SFG) | 408 | 3.44 | -16,54,37 |
| Right occipital (lingual gyrus) | 405 | 3.38 | 20,-85,-8 |
| Left frontal (SFG) | 132 | 3.34 | -21,-3,51 |
| Left temporal (posterior MTG) | 44 | 3.28 | -45,-61,4 |

**Supplementary Table 3**. Regions where FA correlated with working memory performance (uncorrected p=0.001, age as covariate, 10 voxel clusters = 10 mm^3^)

*Healthy controls (positive correlation)*

| **Region** | **Cluster size (mm^3^)** | **Z score** | **MNI coordinates** |
| --- | --- | --- | --- |
| Right ITG | 33 | 3.54 | 49,-7,-35 |
| Left SFG | 14 | 3.48 | -17,35,45 |
| Left SFG | 15 | 3.42 | -22,-8,62 |

*Healthy controls (negative correlation)*

| **Region** | **Cluster size (mm^3^)** | **Z score** | **MNI coordinates** |
| --- | --- | --- | --- |
| Left superior parietal lobe / SLF | 28 | 3.53 | -34,-51,55 |

*Left HS (positive correlation)*

| **Region** | **Cluster size (mm^3^)** | **Z score** | **MNI coordinates** |
| --- | --- | --- | --- |
| Right MFG | 59 | 3.79 | 25,8,50 |
| Right ITG / fusiform gyrus | 89 | 3.46 | 42,-8,-33 |
| Left MFG | 96 | 3.41 | -21,47,-6 |
| Left inferior parietal lobe (angular gyrus / SLF) | 43 | 3.40 | -40,-62,29 |
| Right posterior cingulum | 11 | 3.35 | 5,-29,43 |
| Right posterior cingulum | 37 | 3.34 | 7,-56,54 |
| Right inferior parietal lobe | 31 | 3.27 | 34,-68,37 |

*Left HS (negative correlation)*

| **Region** | **Cluster size (mm^3^)** | **Z score** | **MNI coordinates** |
| --- | --- | --- | --- |
| Left frontal (paracentral lobule) | 22 | 3.55 | -10,-30,71 |
| Left parietal (postcentral) | 31 | 3.32 | -45,-24,58 |

*Right HS (positive correlation)*

| **Region** | **Cluster size (mm^3^)** | **Z score** | **MNI coordinates** |
| --- | --- | --- | --- |
| Right precuneus / cingulum | 300 | 4.06 | 13,-70,43 |
| Left inferior parietal lobe (supramarginal / SLF) | 47 | 4.01 | -52,-36,29 |
| Left posterior MTG / R ILF | 30 | 3.88 | -52,-18,-14 |
| Right anterior cerebellum | 423 | 3.79 | 10,-56,-35 |
| Left anterior cerebellum | 219 | 3.71 | -9,-55,-37 |
| Left posterior STG / R ILF | 29 | 3.41 | -56,-20,-2 |
| Left anterior PHG / cingulum | 19 | 3.32 | -28,-22,-22 |
| Left anterior STG / R ILF | 49 | 3.24 | -51,-6,-10 |
| Left posterior cerebellum | 20 | 3.24 | -5,-55,-46 |

*Right HS (negative correlation)*

| **Region** | **Cluster size (mm^3^)** | **Z score** | **MNI coordinates** |
| --- | --- | --- | --- |
| Right posterior cerebellum | 15 | 3.20 | 28,-58,-53 |

**Supplementary Table 4**. Regions where MD correlated with working memory performance (uncorrected p=0.001, age as covariate, 10 voxel clusters = 10 mm^3^)

*Healthy controls (positive correlation)*

| **Region** | **Cluster size (mm^3^)** | **Z score** | **MNI coordinates** |
| --- | --- | --- | --- |
| Right precuneus / occipital | 50 | 3.43 | 17,-77,44 |

*Healthy controls (negative correlation)*

| **Region** | **Cluster size (mm^3^)** | **Z score** | **MNI coordinates** |
| --- | --- | --- | --- |
| None |  |  |  |

*Left HS (positive correlation)*

| **Region** | **Cluster size (mm^3^)** | **Z score** | **MNI coordinates** |
| --- | --- | --- | --- |
| None |  |  |  |

*Left HS (negative correlation)*

| **Region** | **Cluster size (mm^3^)** | **Z score** | **MNI coordinates** |
| --- | --- | --- | --- |
| Left SFG (medial) / cingulum | 90 | 3.94 | -10,25,55 |
| Right superior parietal lobe / R IFOF | 57 | 3.57 | 19,-70,52 |
| Left superior parietal lobe / L SLF | 25 | 3.38 | -21,-63,56 |
| Right frontal (orbitofrontal) | 15 | 3.38 | 29,28,-20 |
| Right cingulum | 34 | 3.31 | 5,-58,43 |
| Left parietal (postcentral) | 17 | 3.31 | -60,-12,22 |
| Right frontal (orbitofrontal) | 16 | 3.24 | 30,54,-8 |

*Right HS (positive correlation)*

| **Region** | **Cluster size (mm^3^)** | **Z score** | **MNI coordinates** |
| --- | --- | --- | --- |
| None |  |  |  |

*Right HS (negative correlation)*

| **Region** | **Cluster size (mm^3^)** | **Z score** | **MNI coordinates** |
| --- | --- | --- | --- |
| None |  |  |  |
