# Supplementary figures and images for: Structural correlates of impaired working memory in hippocampal sclerosis
Source: Epilepsia. 2013 Apr 24;54(7):1143–53. doi: 10.1111/epi.12193 (PMC3806272; doi:10.1111/epi.12193)

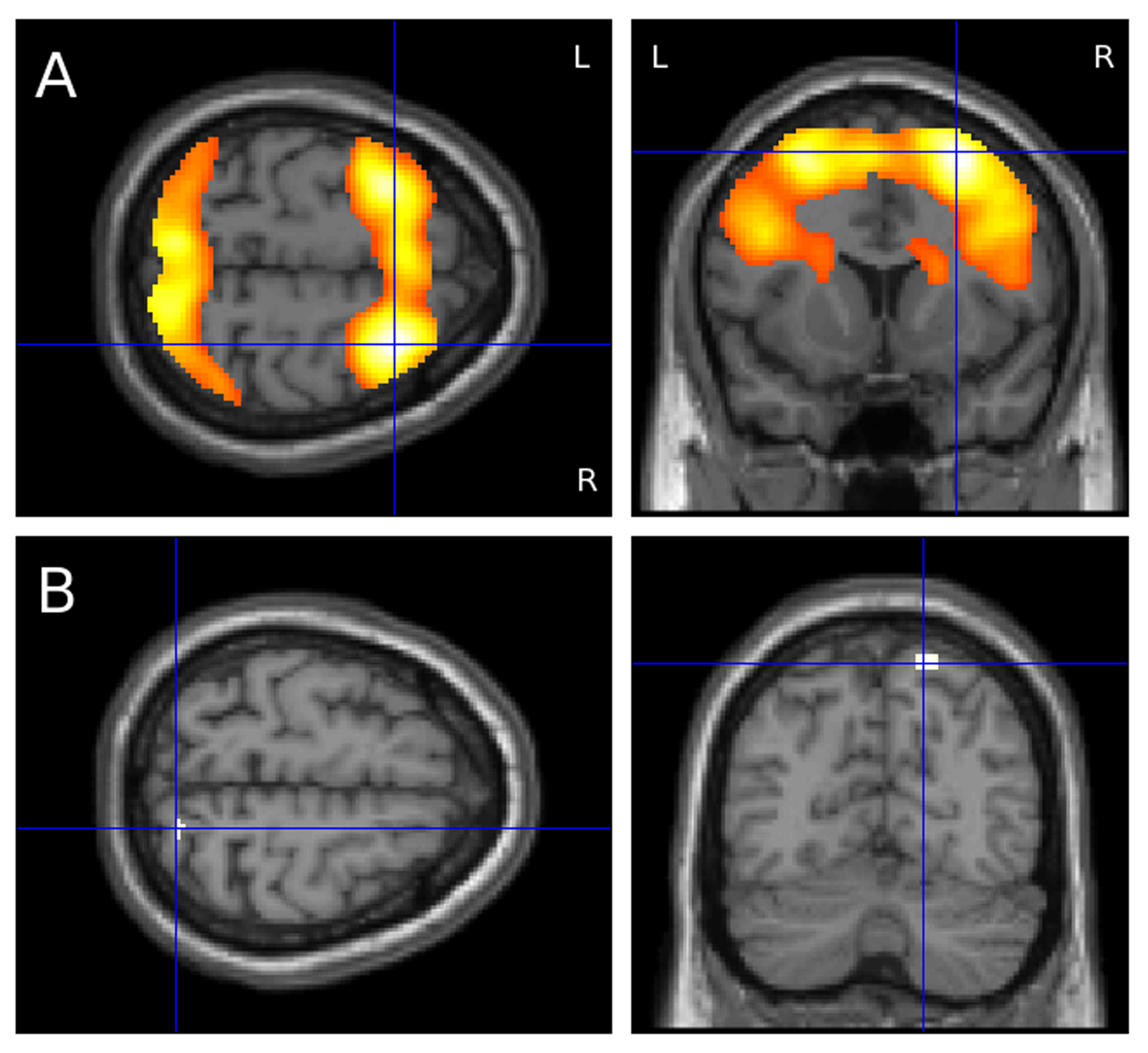

Supplement: Figure S1 — (A) A bilateral frontoparietal working memory network was identified using the 2-back minus 0-back contrast in all subjects. (B) In both LHS and RHS groups there was significantly less activation in the right SPL compared to controls (uncorrected p = 0.001, conjunction). [file epi0054-1143-sd2.tif]

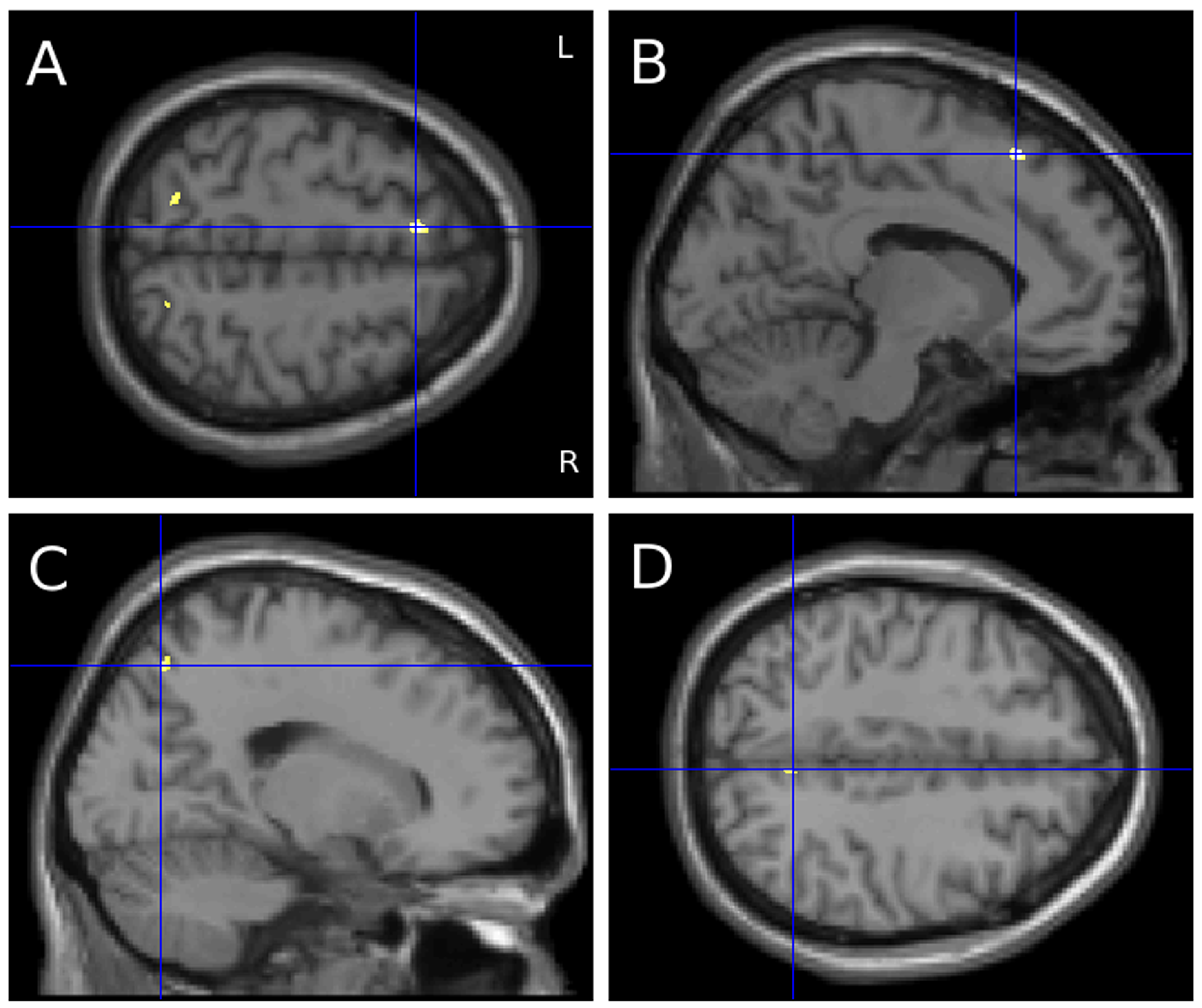

Supplement: Figure S2 — In LHS, better working memory performance correlated with lower MD in the frontoparietal working memory network (left SFG, A and B; bilateral SPL, A and C), the right cingulum (D), and the right orbitofrontal cortex (not shown). [file epi0054-1143-sd3.tif]
